# Calorimetric measurement of energy and nutrient stimulation of microorganisms from the continental deep subsurface

**DOI:** 10.3389/fmicb.2024.1455594

**Published:** 2024-12-23

**Authors:** Jayme Feyhl-Buska, Fabai Wu, Isaiah E. Smith, Douglas E. LaRowe, Alberto Robador, Brittany Kruger, Magdalena R. Osburn, Jan P. Amend

**Affiliations:** ^1^Department of Earth Sciences, University of Southern California, Los Angeles, CA, United States; ^2^Department of Biological Sciences, University of Southern California, Los Angeles, CA, United States; ^3^Division of Hydrologic Sciences, Desert Research Institute, Las Vegas, NV, United States; ^4^Department of Earth and Planetary Sciences, Northwestern University, Evanston, IL, United States

**Keywords:** calorimetry, deep biosphere, Sanford Underground Research Facility, nutrient limitation, microbial activity

## Abstract

Microbial activity in the deep continental subsurface is difficult to measure due to low cell densities, low energy fluxes, cryptic elemental cycles and enigmatic metabolisms. Nonetheless, direct access to rare sample sites and sensitive laboratory measurements can be used to better understand the variables that govern microbial life underground. In this study, we sampled fluids from six boreholes at depths ranging from 244 m to 1,478 m below ground at the Sanford Underground Research Facility (SURF), a former goldmine in South Dakota, United States. The heat produced by microorganisms in these samples was measured in a nanocalorimeter as a proxy for activity. Heat flow measurements on unamended groundwater samples from five of the six boreholes comprising the Deep Underground Microbial Observatory (DeMMO) fell below the limit of detection, suggesting very low metabolic rates. Fluid samples from the borehole that registered a heat signal (DeMMO 6) from 1,478 m deep, were amended with a series of electron donors, electron acceptors, and amino acids before being introduced into the calorimeter. The addition of formate resulted in more than a ~500 nW increase in heat flow relative to the signal for unamended fluids during the first 100 h of incubation while the next highest heat flow arose from nitrate and acetate co-addition, at ~125 nW. Notably, both amendment conditions led to a ~1.5 orders of magnitude increase in cell density without causing major changes to community composition, suggesting that these electron donors and acceptors may be exploited by these communities *in-situ*. The addition of ~0.4 mM casamino acids resulted in a total heat flow of 2.25 μW within 35 h and a more than three orders of magnitude increase in cell density. In these experiments, *Hydrogenophaga* grew to dominate the amino acid amended borehole fluids. The strong microbial response to amino acid addition indicates a deep continental surface community that is limited by the availability of amino acids. A high potential for amino acid metabolism was proposed in genomic studies from this and similar sites but has not been shown in actively growing communities.

## 1 Introduction

Despite being Earth’s largest microbial habitat, the subsurface remains understudied due to its inaccessibility (Whitman et al., 1998; Amend and Teske, 2005; Kallmeyer et al., 2012; Edwards et al., 2012; Colwell and D’Hondt, 2013; McMahon and Parnell, 2014). Microbes in the deep continental subsurface respire orders of magnitude slower than their surface-dwelling analogs (Onstott et al., 1999; Price and Sowers, 2004), and catabolize up to a million times slower than laboratory cultures grown in nutrient-rich media (Hoehler and Jørgensen, 2013). This leads to calculated doubling times, ranging from hundreds to thousands of years (Phelps et al., 1994; Jørgensen and D’Hondt, 2006; Lin et al., 2006).

Determining the catabolic reactions catalyzed by such slow-growing microbes is challenging, as many typical metabolic rate measurements are not designed to detect extremely low levels of activity. For example, measuring microbial turnover rates during incubations (Hoehler and Jørgensen, 2013) is not applicable to the extremely slow metabolic processes characteristic of subsurface systems (Parkes et al., 2005). Furthermore, interpreting the results of such lab-based experiments is often complicated by shifts in the community resulting from selective growth under incubation conditions. Metatranscriptomic analysis of the microbial community RNA provides an alternative option to assess microbial activity in the subsurface. This approach, however, is hampered by difficulties with RNA extraction (Schippers et al., 2005; Biddle et al., 2006; Orsi et al., 2013; Urich et al., 2014; Pachiadaki et al., 2016; Zinke et al., 2017) and limited by the quantity of *in-situ* activity.

To overcome these limitations, we used a nanocalorimeter to measure the heat associated with microbial activity in underground samples collected from the Sanford Underground Research Facility (SURF). Calorimetry allows for the agnostic measurement of heat flow and therefore records the total change in enthalpy for all reactions occurring in a system, biotic and abiotic. Additionally, this technique boasts a low detection limit, ~1.2 nW/mL (Braissant et al., 2010). Nanocalorimetry can measure potential bacterial heat production in cell cultures of approx. 1 × 10^2–3^ cells/mL (Higuera-Guisset et al., 2005; Braissant et al., 2010; Robador et al., 2018) and natural samples of 1 × 10^3–4^ cells/mL (Robador et al., 2016). Cell densities in SURF samples are well within this range, between 1 × 10^3^ cells/mL and 4 × 10^5^ cells/mL. Furthermore, the sum of the total heat over time was complemented with concentration measurements of products and reactants evolving during the experiment to thermodynamically constrain the reactions being catalyzed. For example, Robador et al. (2018) used this technique to identify microbial lactate oxidation as the key reaction in a system where multiple microbial metabolic pathways could be employed. Prior studies at SURF revealed discrepancies in metabolic predictions at identical boreholes, noting that while sulfate and nitrate reduction pathways were frequently identified, they were not the most energetically advantageous (Osburn et al., 2014; Momper et al., 2017). Further analysis of functional genes across DeMMO sites by Momper and colleagues in 2023 showed that genomic potential did not align with the predominant geochemical species at each location. However, the 2023 study also reported ubiquitous potential for the metabolism of small organic molecules, such as amino acids and acetate. This study aims to identify which metabolic pathways are actively employed by microbes, focusing on their actual metabolic activities instead of their potential capabilities.

## 2 Materials and methods

### 2.1 Sample collection and geochemical characterization

Samples were collected from all the Deep Mine Microbial Observatory (DeMMO; Osburn et al., 2019) at the Sanford Underground Research Facility (SURF) in Lead, South Dakota, United States. SURF is located within the former Homestake Gold Mine and DeMMO accesses groundwater from six separate areas within SURF, ranging in depth from 244 m to 1,478 m beneath the surface. Prior research utilizing the DeMMO infrastructure has developed a geochemical and biological time series (Osburn et al., 2019; Osburn et al., 2020), and explored the metabolic capabilities at SURF through *in-situ* cultivation and studies on energetics and metagenomics (Osburn et al., 2014; Momper et al., 2017; Casar et al., 2021a; Casar et al., 2021b; Momper et al., 2021; Momper et al., 2023).

The samples retrieved for this study were obtained in tandem with those of long-term monitoring for geochemical and biological variability within the DeMMO network (Osburn et al., 2019). All six DeMMO boreholes were sampled (DeMMO 1–6) during two expeditions, on December 5–10th, 2016, and September 9–14th, 2018. Data from the time of sampling is available in Table 1. Detailed sampling procedures for geochemical and community analysis are available in companion studies (Osburn et al., 2019; Osburn et al., 2020). As described in these studies, geochemical analysis was performed on samples either directly at the field site or after being prepared and shipped to the laboratory for more comprehensive processing. At the field, temperature, pH, redox-sensitive ions, oxidation reduction potential (ORP), and conductivity were measured using a Hach field testing kit (Hach, Loveland, CO, United States) and a Myron Ultrameter II (Myron L Company, Carlsbad, CA, United States). Additional geochemical analyses were carried out in other academic or commercial labs: major dissolved cations and anions (ACZ Laboratories, Steamboat Springs, CO, United States), dissolved organic carbon (DOC; Anatek Labs, Moscow, ID, United States) and dissolved inorganic carbon (DIC; Northwestern Stable Isotope Biogeochemistry Laboratory, Evanston, IL, United States). Dissolved gasses were measured via gas chromatography on a Shimadzu GC-2014 using FID and TCD detectors and referenced to Scott analytical standards (Air Liquide, Paris, France). Cells from field samples were fixed with paraformaldehyde and stored at −20°C prior to staining with DAPI (4′,6-diamidino-2-phenylindole) and concentration onto a 0.22 μm black polycarbonate filter (Millipore, Burlington, MA).

**Table 1:** Geochemical concentrations at six DeMMO boreholes during two sampling trips.

Date of Sampling	Borehole	Depth (m)	Temperature (°C)	pH	ORP (mV)	Conductivity (uS)	Total Dissolved Solids (ppm)	Nitrate (mg/L)	Ammonia (mg/L)	Ferrous Iron (mg/L)	Sulfide (ug/L)	Sulfate (mg/L)	Dissolved Oxygen (mg/L)	Dissolved Organic Carbon (mg/L)
December, 2016	1	244	10	7.37	−96	956.1	688.7	0.2	0.07	2.27	0	297	0	0.4
	2	244	12.5	7.72	−93	626.5	443.5	0.1	0.03	0.31	29	156	0.54	0.387
	3	610	16.2	7.27	−30	3,036	2,313	0.2	0.28	2.92	0	1,540	0.147	0.259
	4	1,250	22.6	8.88	−278	1774	1,296	1	1.56	0.01	537	262	0.043	0.174
	5	1,478	31.4	9.07	−233	1,540	1,109	0.7	0.46	0	329	80.3	0.058	BDL
	6	1,478	24.6	8.64	−205	7,978	6,685	0.4	0.08	1.23	55	4,370	0.09	0.136
September, 2018	1	244	10.6	NA	−78	980.3	701.1	0.3	0.08	2.68	0	360	0.042	0.42
	2	244	12.3	NA	−49	623.8	440.6	0.2	0.03	0.31	0	97.4	NA	0.381
	3	610	16.2	NA	−77	3,112	2,370	0.2	0.17	1.8	17	1740	0.009	0.244
	4	1,250	22.4	NA	−207	1781	1,302	0.9	1.36	0	780	291	0.032	0.181
	5	1,478	31.4	NA	−185	1,537	1,105	0.9	0.46	0	334	173	0.121	0.173
	6	1,478	21.5	NA	−194	7,873	6,561	0.3	0.12	0.88	38	4,380	0	0.252

“BDL” indicates that the constituent was “below detection limit,” “NA” indicates that the measurement is not available from that date.

### 2.2 Calorimetry

Groundwater samples for calorimetry were collected in sterile, anaerobic, glass serum bottles. Prior to fieldwork, the bottles were combusted at 400°C for 4 h, closed with butyl stoppers and seals, then flushed with nitrogen gas until anaerobic. The anaerobic combusted bottles were autoclaved at 121°C for 30 min, prior to being evacuated to maximum achievable negative pressure. In the field, the tops of the prepared serum bottles were sterilized with 70% isopropanol wipes, and Viton^®^ tubing (DuPont Performance Elastomers LLC, Wilmington, DE, United States) and needles were used to anaerobically and aseptically transfer fluids from the boreholes to the bottles. For each borehole, at least one serum bottle was filled with borehole water that first passed through a 0.2 μm filter. A few weeks after samples were returned to the lab, these filtered borehole water samples were stained with acridine orange and collected onto a 0.22 μm black polycarbonate filter (Millipore, Burlington, MA) prior to visualization on a Zeiss Axiovision Epifluorescent Microscope to inspect for growth indicative of contamination, but no cells were observed in any of the filtered water bottles.

All borehole fluids were kept at 4°C following sample collection until placed in the calorimeter. Samples for calorimetry were prepared as described by Robador et al. (2016), with slight modifications. Briefly, 4 mL TA Instruments ampules were sealed with aluminum foil and combusted at 400°C for 4 h (TA Instruments, New Castle, DE, United States). Combusted ampules were then autoclaved at 121°C for 30 min. Groundwater was aseptically and anaerobically removed from the serum bottles collected in the field and 2 mL was transferred into each ampule in a Coy Laboratories anaerobic chamber (Grass Lake, MI, United States) at <5 ppm oxygen. Ampules were sealed with a crimp top cap, which includes a built-in rubber stopper. For the experiments that included amendments, the added chemical was mixed with the groundwater aseptically in the anaerobic chamber prior to being transferred into the calorimetric ampule.

For all calorimetric analyses, a TAM III thermal activity monitor (TA Instruments-Waters LLC, New Castle, DE, United States) equipped with two nanocalorimeters was equilibrated to 28°C prior to the start of experiments. Reference ampules, calorimetric samples identical in chemical composition to the groundwater sample ampules, but with biomass removed via a 0.1 μm filter, were loaded into the reference position in each calorimeter prior to the beginning of each new experiment. Once the reference ampules equilibrated with the calorimeters, samples were lowered into the sample slots on each calorimeter, allowing for thermal equilibration halfway through the lowering process into measuring position.

**Figure 1:**
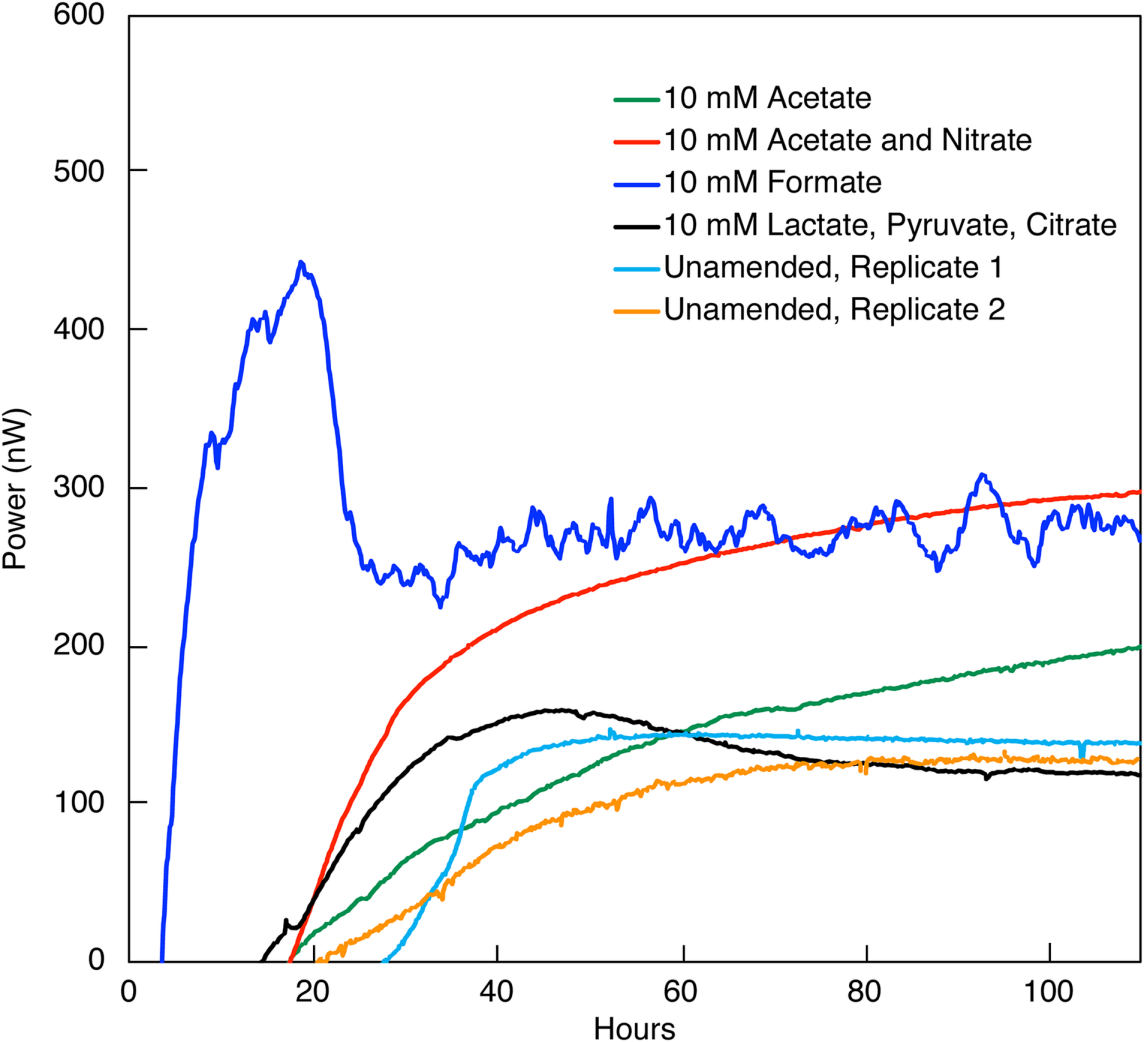
Heat flow (Power) from the indicated amendments to DeMMO 6 borehole fluid. The baseline heat flow signal has been corrected using the synchronization method.

**Figure 2:**
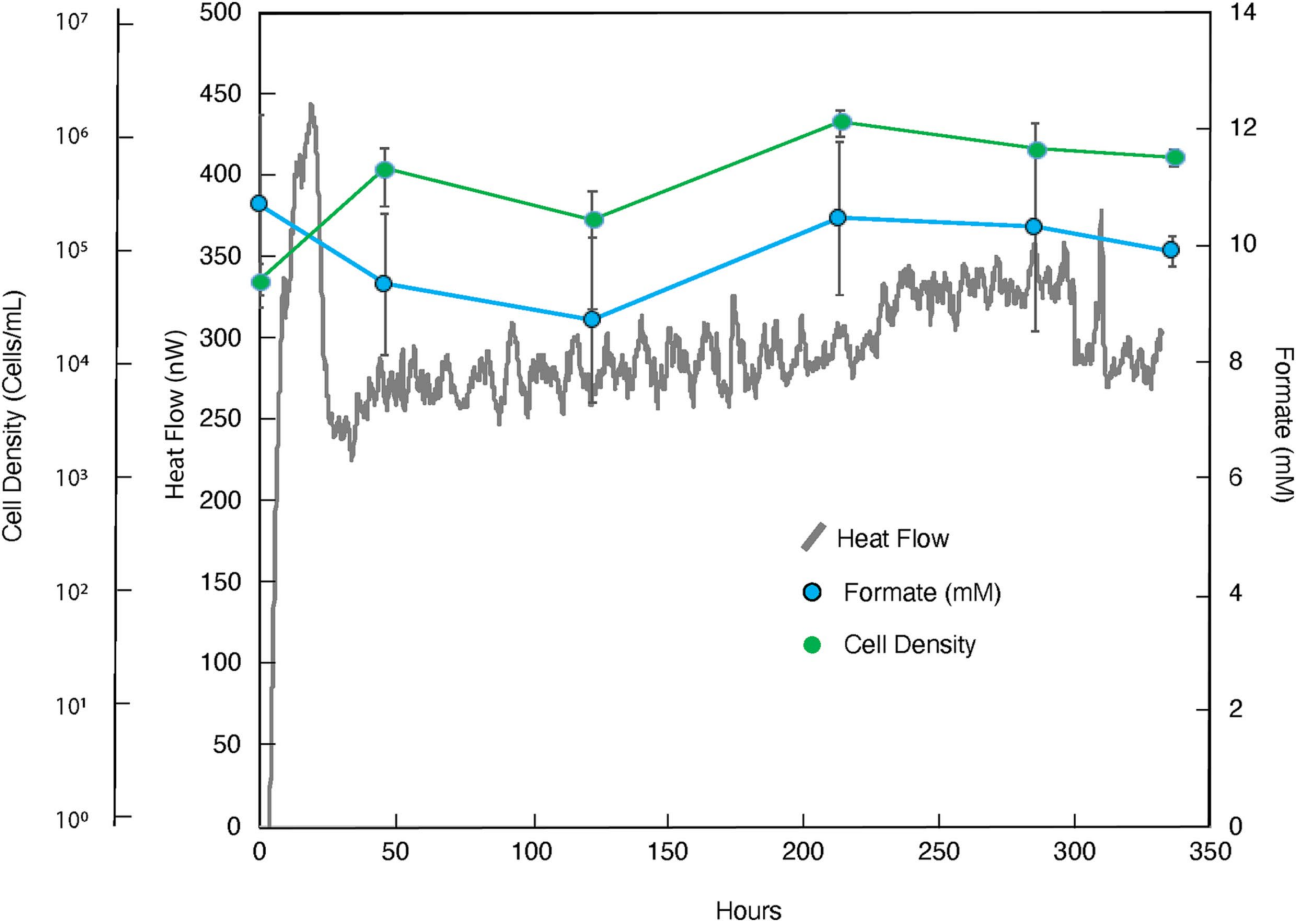
Heat flux (Power), cell density and formate concentration as a function of time for the amendment experiment in which formate (10 mM) was added to borehole fluids from DeMMO 6. The baseline heat flow signal has been corrected using the synchronization method.

**Figure 3:**
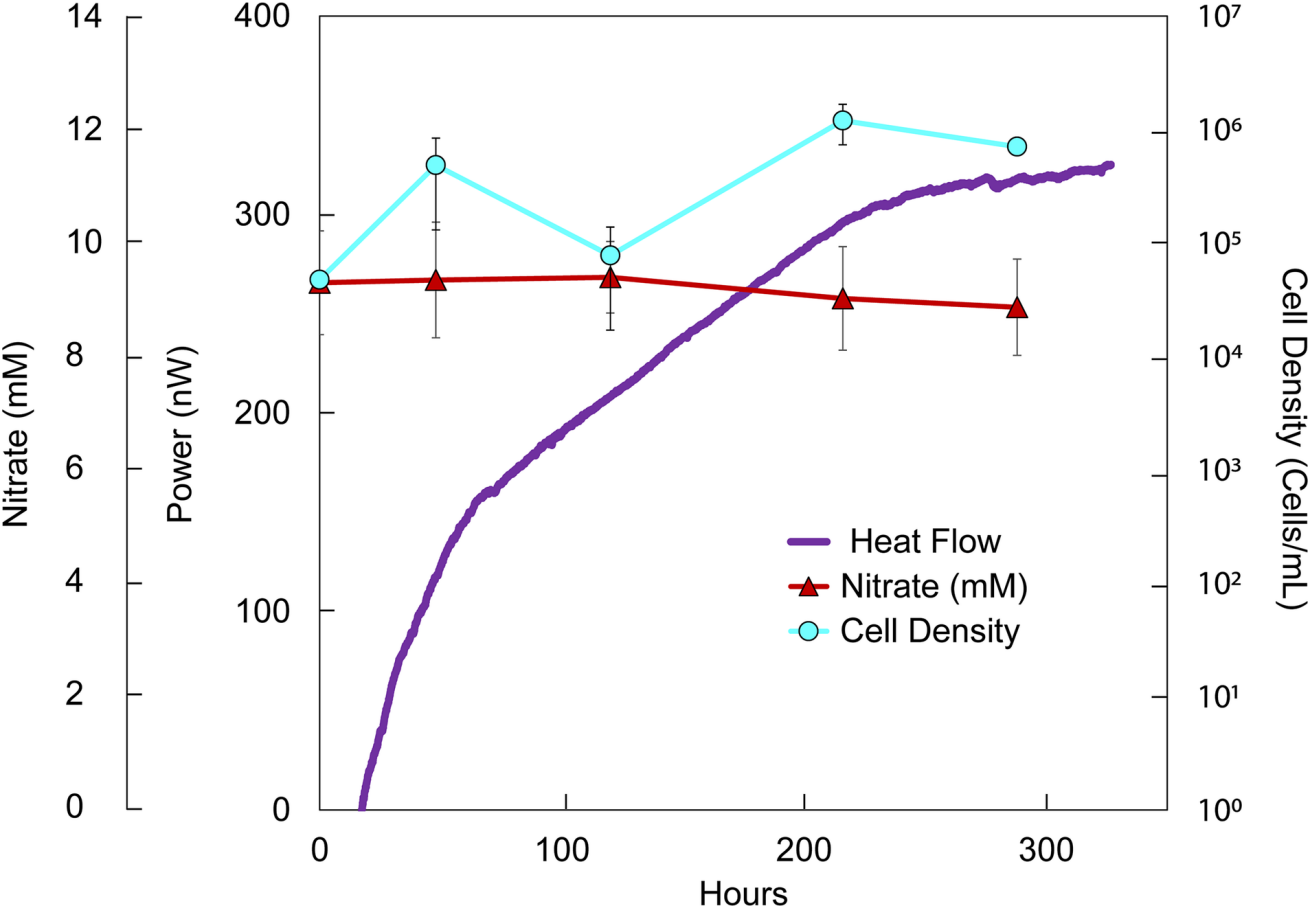
Heat flux (Power), cell density and nitrate concentration over time in DeMMO 6 fluids amended with nitrate (10 mM) and acetate (10 mM). The baseline heat flow signal has been corrected using the synchronization method.

**Figure 4:**
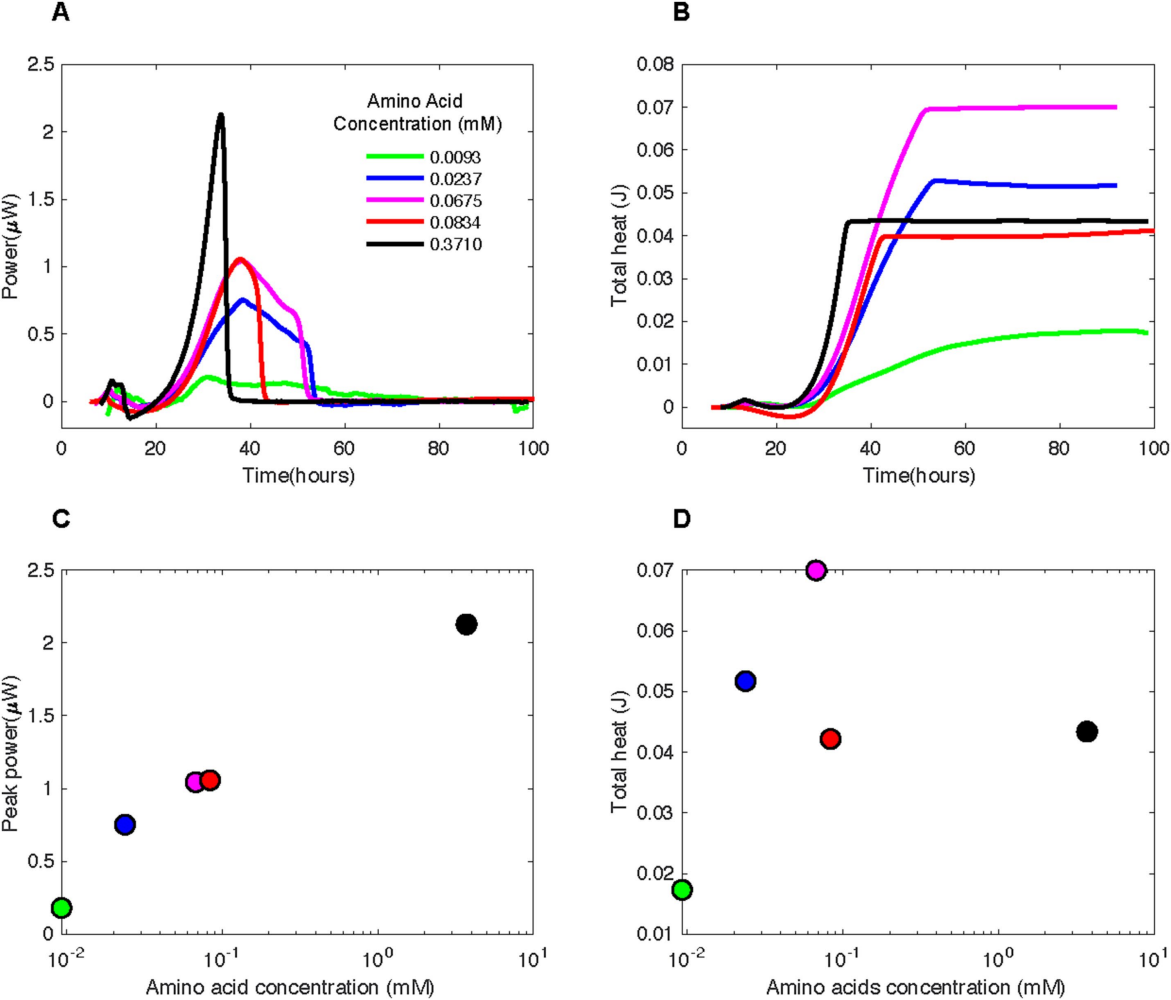
**(A)** Heat flux (Power) and **(B)** cumulative heat as a function of time and **(C)** peak power and **(D)** total heat as a function of amino acid concentrations associated with experiments in which various concentrations of casamino acids were added to borehole fluids from DeMMO 6. The baseline heat flow signal has been corrected using the power function method.

**Figure 5:**
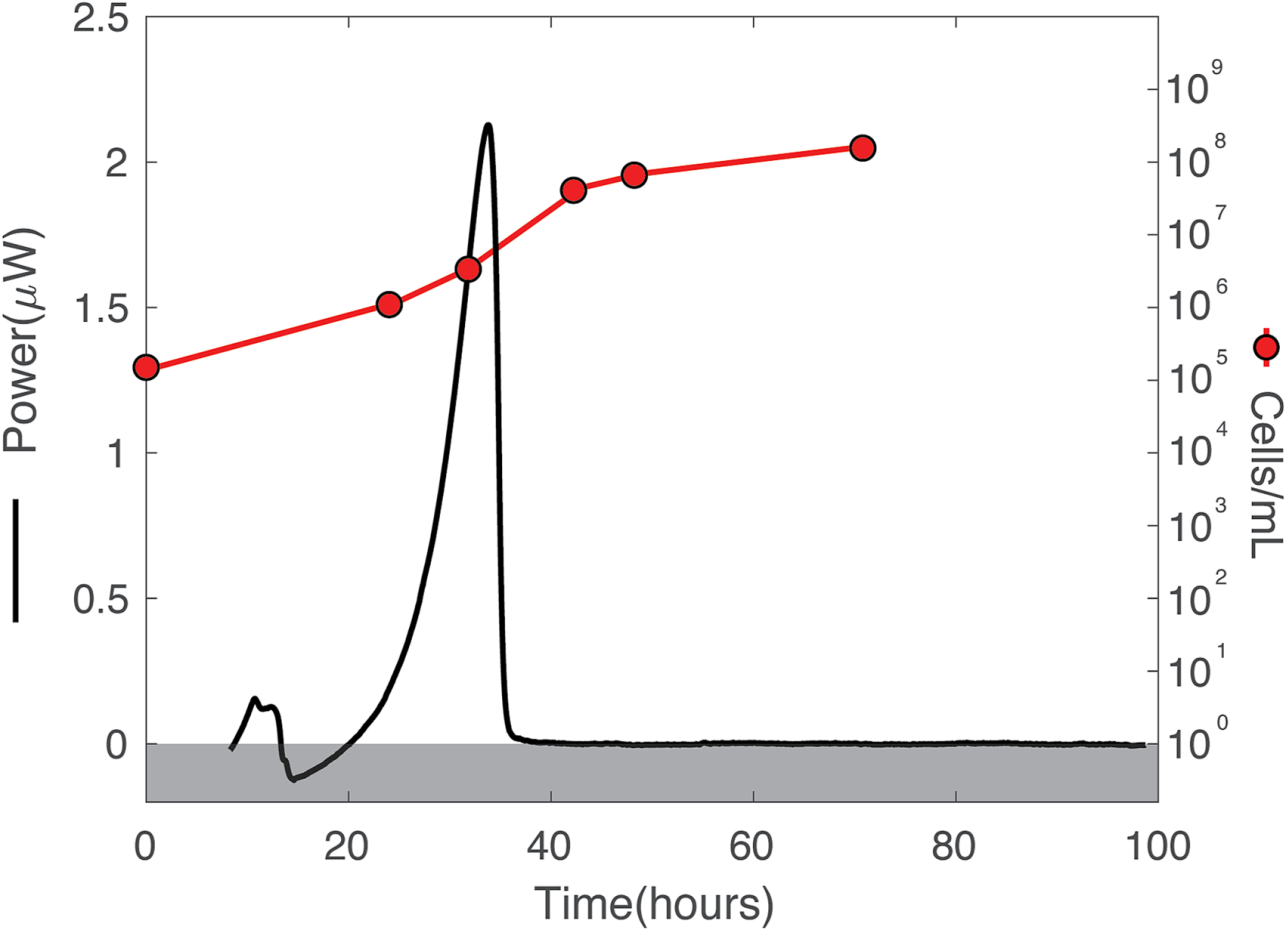
Heat flux (Power) and cell density over time in DeMMO 6 fluids amended with 0.3710 mM amino acids. The baseline heat flow signal has been corrected using the power function method. A gray box covers all data below 0 to facilitate ease of understanding for the cell/mL axis, which only starts above 0.

Due to the high system sensitivity, it is necessary to adjust the experimental data to compensate for the heat produced when lowering experimental ampules into the calorimeter’s measuring position. In this manuscript, two methods were utilized for the removal of perturbation signal. For low total-signal samples (such as in Figures 1–3), the “synchronization method” was used, which involved synchronizing the heat signals across all experiments to make the initial disruption in each experiment’s heat curve easily recognizable. Only data collected after this initial surge of heat are presented; any data collected before this convergence point are deemed artifacts resulting from the insertion process and are thus disregarded. In the amino acid amended experiments (such as in Figures 4, 5), microbial activity generated a significant heat signal, allowing for the use of the less human-biased “power function method.” This permitted the use of a power function (f(x) = a*xb + c) to model the artificial heat effects. The function helped identify the initial and final data segments as background noise due to disturbances resulting from the insertion of the sample, which were then excluded from the analysis. An illustration of this method can be found in Supplementary Figure 1. A variety of negative controls were tested to subtract out the potential confounding factors in quantification. To assess whether the 0.1 μm filtered borehole fluid in the reference ampule was not, in fact, sterile, reference ampules were prepared with borehole fluid autoclaved once, twice, and once with additional 0.1 μm filtration. Different methods to reduce baseline heat signal introduced during sample-insertion process were also tested. The duration of time that the sample was allowed to equilibrate during lowering was varied, as was the time the sample was given to return to 28°C after storage at 4°C. Based on this test work, the methodology described earlier in this section for reference and sample ampule handling was selected.

Amendments to the calorimetric samples were prepared as concentrated, anaerobic, sterile solutions. Borehole water samples and amendment solutions were combined aseptically in an anaerobic chamber, at a final concentration of 10 mM for all additions except the amino acid and ammonium additions. Casamino Acids (Amresco Bacterological Grade) were utilized for the amino acid addition experiments and amended into the borehole fluids as a sterile anerobic solution at varying percentages. These percentages were later converted into molarities using the average molecular weight for casamino acids, 539.583 g/mol, given by the manufacturer. It should be noted that all “amino acid” concentrations reported here are a combination of free amino acids and small peptides that result from the digestion of casein. Therefore, tryptophan is not present in this mixture, and the remaining amino acids are not present in equal abundances. Electron donor (ED) and acceptor (EA) additions were predominantly conducted individually, with the assumption that the amended ED or EA would be coupled with a redox partner from the borehole fluid. Exceptions to this method were made for the co-addition of acetate and nitrate, while three potential EDs were added simultaneously with lactate, citrate, and pyruvate. Following the conclusion of all calorimetric experiments, ampules were closely examined for cracks in the glass and gaps in the seal, to ensure that vial integrity was maintained throughout the experiment.

### 2.3 DNA and RNA processing

While in the field, samples for genomic DNA and RNA extraction were collected onto 2–4 sterile replicate housing-encased 0.1 μm Supor filters per borehole. Based on cell enumeration from fluids and fluid rates, a minimum of 10^6^ cells were captured onto each filter, with samples collected in <1 h (Table 2). Most filtration was completed within 40 min, and two filters contained 10^9^ cells. Collected cells were immediately frozen at −80°C and kept at this temperature until extraction.

**Table 2:** Total cells collected on filters from the six DeMMO (Deep Mine Microbial Observatory) boreholes considered in this study.

Borehole	Filter	Total filtered (mL)	Duration of filtration (min)	Cell density at borehole (cells/mL)	Total cells on filter
1	1	7,286	40	1.10 × 10^3^	8.03 × 10^6^
	2	10,615	40	1.10 × 10^3^	1.17 × 10^7^
2	1	6,077	40	5.63 × 10^3^	3.42 × 10^7^
	2	1,282	40	5.63 × 10^3^	7.22 × 10^6^
3	1	1,241	40	3.13 × 10^3^	3.88 × 10^6^
	2	520	40	3.13 × 10^3^	1.63 × 10^6^
	3	780	40	3.13 × 10^3^	2.44 × 10^6^
4	1	1,240	40	6.06 × 10^3^	7.51 × 10^6^
	2	640	40	6.06 × 10^3^	3.88 × 10^6^
	3	820	40	6.06 × 10^3^	4.97 × 10^6^
5	1	1,320	40	5.60 × 10^3^	7.40 × 10^6^
	2	3,640	40	5.60 × 10^3^	2.04 × 10^7^
	3	160	40	5.60 × 10^3^	8.96 × 10^5^
	4	10,000	40	5.60 × 10^3^	5.60 × 10^7^
6	1	900	20	4.03 × 10^5^	3.63 × 10^8^
	2	2,720	20	4.03 × 10^5^	1.10 × 10^9^
	3	100	20	4.03 × 10^5^	4.03 × 10^7^
	4	6,844	20	4.03 × 10^5^	2.76 × 10^9^

Borehole water can be split into spigots of variable flow rate; here, flow rate was measured every 10 min and has been averaged to determine the “Total Filtered” water for each borehole. The total cells on filter are calculated from the cell densities and the total fluids filtered.

Initial DNA and RNA extractions from the filters employed a modified version of the protocol in Momper et al. (2017), which was used for genomic DNA extraction from the same sample site. In short, filters were subjected to physical and chemical lysis through a combination of bead beating, freeze/thaw cycles, and sequential exposure to an extraction buffer (Tris and EDTA) and a TESC buffer. Following cell lysis, proteins, humic acids, and other contaminants were removed via 24:1 chloroform:isoalcohol precipitation. The resulting pellet was cleaned using 100% ethanol, prior to being dried and re-suspended in TE buffer. Following DNA and RNA extraction, eluents were divided in half, and in one aliquot, DNA was quantified on a Qubit 2.0 Flurometer (Invitrogen, Carlsbad, CA) using a Qubit dsDNA HS kit. In the other aliquot, RNasein (Promega, Madison, WI, United States) was added to prevent RNA degradation, and TURBO DNase (Invitrogen, Carlsbad, CA, United States) was utilized to remove genomic DNA. RNA was then quantified using a Qubit RNA HS kit. cDNA synthesis was accomplished with SuperScript III First-Strand Synthesis SuperMix (Invitrogen, Carlsbad, CA) using random hexamers, according to manufacturer protocols.

RNA was also extracted using a RNeasy PowerBiofilm Kit (Qiagen, Hilden, Germany), following manufacturer protocols, except that DNase 1 solution was not added to the reactions until after the protocol was completed, allowing for simultaneous DNA and RNA extraction. Once extracted, RNA resulting from this procedure was treated in the same manner as RNA resulting from the procedure noted above.

DNA for 16S rRNA gene community comparisons between the calorimetric experiments was extracted using a DNEasy Power Biofilm Kit (Qiagen, Hilden, Germany), following manufacturer protocols, and quantified with a Qubit dsDNA HS kit. 16S rRNA gene amplification utilized the 515F/926R primer pair (Parada et al., 2016) and GoTaq reaction mix (Promega, Madison, WI, United States), while in a Veriti 96 Well Thermal Cycler for the following steps: 95°C for 3 min, 30 cycles of 95°C for 45 s, 50°C for 45 s, and 72°C for 1 min 30 s. The presence and quality of PCR products was confirmed via gel electrophoresis. PCR products were shipped to Molecular Research DNA (Shallowater, TX, United States) for indexing, purification with Ampure XP beads, normalization and pooling prior to paired end sequencing of the 16S rRNA gene on an Illumina MiSeq platform. The resulting reads were pre-processed in the FASTq Processor (Molecular Research DNA) to remove barcodes, linker primers, and reverse primers. QIIME2, running in R 4.0.1, demultiplexed reads (Bolyen et al., 2019) and the Divisive Amplicon Denoising Algorithm 2 (DADA2) denoised reads (Callahan et al., 2016). This process produced Amplicon Sequence Variants (ASVs), which were compared to the sequences listed in the Ribosomal Database Project’s SILVA v138 Database (Quast et al., 2013; Yilmaz et al., 2014). Relative abundance and diversity plots were made in the phyloseq and ggplot2 packages, running in R (McMurdie and Holmes, 2013; Wickham, 2016).

### 2.4 Viral and prokaryotic quantification

Prokaryotic cells were counted using a Zeiss Axiovision Epifluorescent Microscope. Cells were fixed with paraformaldehyde and stored at −20°C, then stained with acridine orange and collected onto a 0.22 μm black polycarbonate filter (Millipore, Burlington, MA). Viral particles were quantified following Haas et al., 2014. In brief, samples were fixed with paraformaldehyde, filtered onto a 0.02 μm Whatman Anodisc 25 filter, and stained with SYBR Gold. After dye incubation, the excess SYBR Gold was removed and the filter was mounted to a slide using a filtered ascorbic acid/phosphate buffered saline/glycerol mixture. Viral and prokaryotic cells were visualized using a cyan filter and the Image-Pro Plus 7.0 program. 25 micrographs were taken at random and the viral and prokaryotic quantities counted manually.

### 2.5 Parallel experiments

Incubation experiments identical to those carried out in the calorimeter were carried out in parallel in a darkened incubator to determine how the concentrations of cells, anions, organic acids and gases changed with time (samples cannot be removed from the calorimeter during an experiment without significant loss of data). Cell densities and gas concentrations were determined as described above. Anion concentrations were measured on a Metrohm Ion Chromatograph (Metrohm, Riverview, FL, United States) equipped with an anion separation column. Organic acid measurements were made on an Agilent 1,100 Series HPLC (Santa Clara, CA, United States) utilizing an Agilent Hi-Plex H column.

### 2.6 Thermodynamic calculations

Standard state enthalpies of reaction, ∆*H_r_*^0^, were calculated at 28°C and 1 bar using the revised HKF equations of state (Helgeson et al., 1981; Tanger and Helgeson, 1988; Shock et al., 1992), the SUPCRT92 software package (Johnson et al., 1992) and thermodynamic data taken from a number of sources (Amend and Helgeson, 1997; Dick et al., 2006). For the reactions in which the identities of the amino acids were not known, i.e., when Casamino acids were used, a weighted average of ∆*H_r_*^0^ for the 20 common naturally-occurring amino acids in the proportion that they are found in *E. coli* (Neidhardt, 2005) were used to estimate the enthalpies of amino acid oxidation reactions. This approximation was made since the enthalpy calculation requires knowing the defined proportion of amino acids in solution, but the relative amount of each amino acid in Casamino acids is not known. However, the amino acid composition of *E. coli* has been measured. Values of ∆*H_r_*^0^ for the half reactions describing the full oxidation of carbon, but not other elements were combined with values of ∆*H_r_*^0^ for half reactions describing the reduction of O_2_ to H_2_O, SO_4_^2−^ to HS^−^ and NO_3_^−^ to N_2_ to calculate values of ∆*H_r_*^0^ for the oxidation of a nominal amino acid by these three EAs. The enthalpies of reaction were then used in combination with reaction stoichiometries (mol electron acceptor / mol amino acid) and the total heat produced in the calorimeter (0.0433 J) to determine the number of moles of reactants consumed and CO_2_ produced. These results are summarized in Table 3.

**Table 3:** Summary of reactions considered to be sources of heat in the calorimetry experiments carried out on SURF borehole fluids and the cumulative number of moles of specified reactants and CO_2_ in these reactions that would be consumed or produced to generate 0.0433 J of heat, the total amount of heat produced following amendment with 0.371 mM amino acids.

Reaction	Moles consumed	Moles produced (CO_2_)
C_3_H_7_NO_2_ + 2C_2_H_5_NO_2_ + 2H_2_O → CO_2_ + 3CH_3_COO^−^ + 3NH_4_^+^	Alanine: 4.27 × 10^−7^	4.27 × 10^−7^
O_2_ + AA → H_2_O + CO_2_	O_2_: 7.15 × 10^−8^	7.15 × 10^−8^
SO_4_^2−^ + AA → HS^−^ + CO_2_	SO_4_^2-^: 3.97 × 10^−6^	7.94 × 10^−6^
NO_3_^−^ + AA → N_2_ + CO_2_	NO_3_^-^: 9.71 × 10^−8^	1.21 × 10^−7^
0.5O_2(aq)_ + H_2(aq)_ → H_2_O	H_2(aq)_: 1.59 × 10^−7^	NA
SO_4_^2−^ + 4H_2(aq)_ + H^+^ → HS^−^ + 4H_2_O	H_2(aq)_: 7.91 × 10^−7^	NA
NO_3_^−^ + 2.5H_2(aq)_ + H^+^ → 0.5N_2_ + 3H_2_O	H_2(aq)_: 1.70 × 10^−7^	NA

“AA” refers to amino acid and “NA” means not applicable.

## 3 Results

### 3.1 Indications of microbial activity in unamended borehole fluids

Heat signal in unamended DeMMO 1–5 borehole fluids was below detection, at a limit of ~1.2 nW/mL and despite incubation for at least 150 h. None of the possible sources of signal interference that were tested (see Methods section) improved the replicability or detectability of heat flow measurements in these samples. Only DeMMO 6 borehole fluid produced a replicable power signal equilibrating at ~100 nW within 40 h (Figure 1) and was further interrogated, as discussed below.

**Figure 6:**
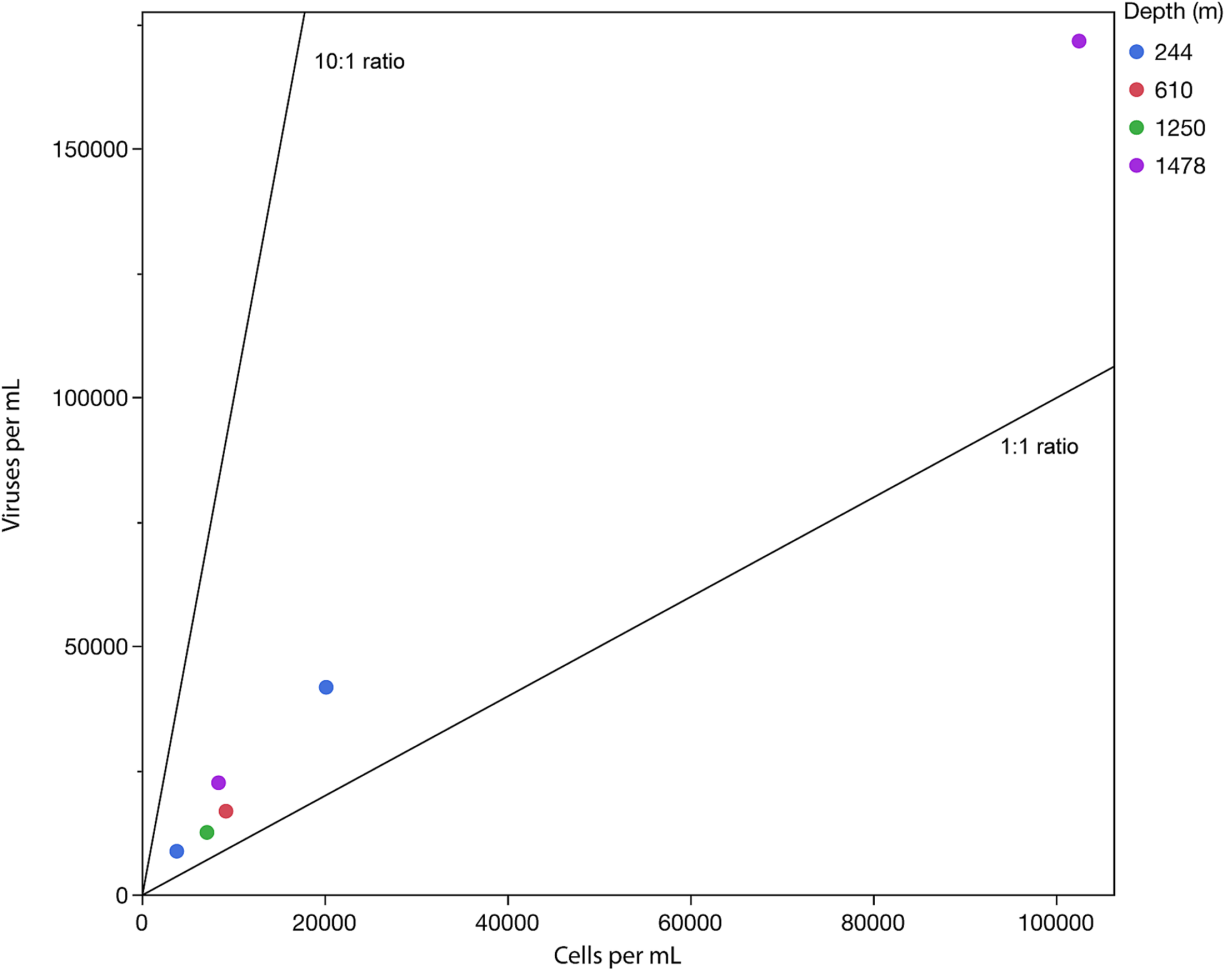
Virus-to-prokaryote ratio (VPR) in samples taken from DeMMO boreholes from the indicated depths below the local surface. The black diagonal lines provide a visual guide to specific VPR values with a base of 10.

Virus-to-prokaryote ratio (VPR) measurements summarized in Figure 6 correlate with the low heat signals measured on the calorimeter. It can be seen in this figure that VPR linearly scaled with cell density, but not with depth within the mine. The 6 DeMMO boreholes access 4 depths at SURF, with DeMMO 6 accessing a borehole containing ~2 orders of magnitude more cells per milliliter than the other DeMMO boreholes (Table 2). The average VPR across the samples was 2.03, with a high at DeMMO 5 (2.66) and low at DeMMO 6 (1.67). DeMMO 5 and 6 are at the same depth in the mine although access very different fluids (Osburn et al., 2019) while boreholes from 244 m deep had similar VPRs (2.25 and 2.06).

mRNA, another proxy for active microbial functions, was extracted from all DeMMO boreholes, but was not detected at a sufficient level for functional interpretation. This result was noted despite the extraction of ample DNA from the same samples and consistently high mRNA recoveries in the positive controls (see Methods section).

### 3.2 DeMMO 6 amendments

#### 3.2.1 Electron acceptors and donors

**Figure 7:**
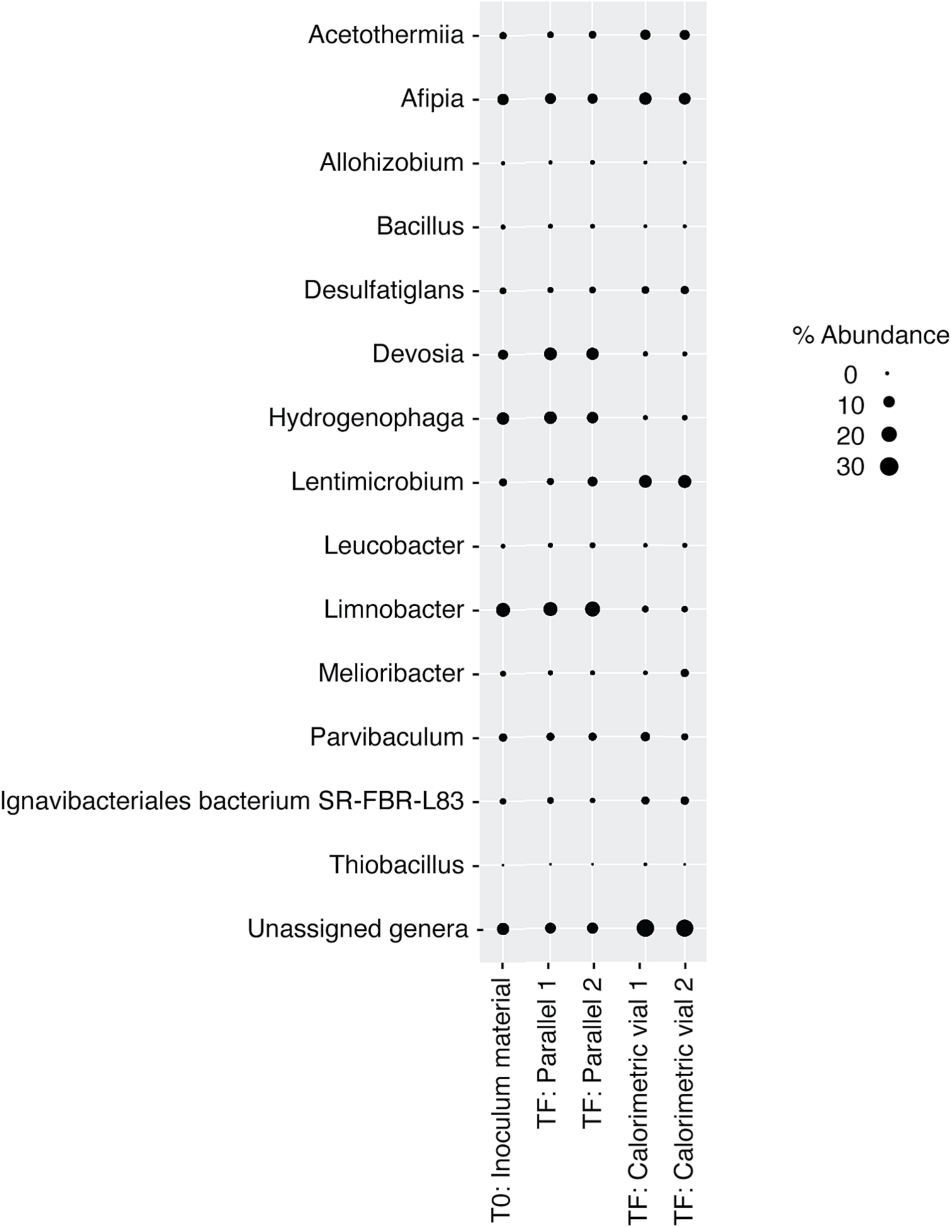
Relative abundance of microbial genera in DeMMO 6 borehole fluids before (*t_0_*) and after (*t_F_*) incubations with lactate, citrate, and pyruvate amendments. “Parallel 1” and “Parallel 2” refer to duplicate incubation experiments run outside of the calorimeter and “calorimetric vial 1” and “calorimetric vial 2” correspond to replicate incubations experiments run inside the calorimeter. “Unassigned genera” indicates taxa for which taxonomy could not be assigned at the genus level.

DeMMO 6 borehole fluid samples were amended with EAs and/or EDs/carbon sources to investigate the genomic potential and energetic potential of microorganisms previously described in DeMMO (Momper et al., 2017; Osburn et al., 2014) but exhibited low increases in heat flow relative to unamended samples (Figure 1). By ~50 h, all samples reached a heat flow signal that was maintained consistently for upwards of 300 h (only data through 100 h shown in Figure 1). Of the amendment experiments, formate (dark blue curve in Figure 1) and an acetate/nitrate mixture (orange curve) increased the heat flow above that for unamended borehole waters (light blue and yellow curves) by ~500 nW and ~ 125 nW, respectively. Fluid sample amended with only acetate (green curve) and fluid sample amended with a mixture of citrate, lactate, and pyruvate (black curve) produced no significant change with respect to unamended fluid samples (Figure 6). In borehole waters amended with a combination of citrate, lactate, and pyruvate, heat signal peaked at ~175 nW around 45 h. Cells density increased from 1.16 × 10^4^ cells/mL to 4.84 × 10^5^ cells/mL over a period of 240 h, although the community composition remained very consistent throughout the duration of the experiment (Figure 7; Supplementary Figure 2). At the beginning of the experiment (*t*_0_), no single genus accounted for >25% of the community, with the most abundant genera identified as *Afipia*, *Devosia*, *Hydrogenophaga* and *Limnobacter*. These same genera remained in high abundance at the end of the experiment, both within the calorimetric vials and within the parallel experiments.

The addition of formate as potential ED and carbon source resulted in a more robust and oscillating heat signal, peaking at ~450 nW at ~20 h before sharply dropping to ~225 nW and tapering off at ~100 nW above this value for the duration of the experiment (336 h; Figure 2; replication in Supplementary Figure 3). Concurrently, cell densities per milliliter increased from 3.99 × 10^4^ to 4.18 × 10^5^ in the first 46 h and remained relatively constant throughout the duration of the experiment. A notable size increase in cell radius, as visualized microscopically, was also noted during this time (data not shown). It can be further seen that, within measurement uncertainty, apparent formate concentrations did not change.

**Figure 8:**
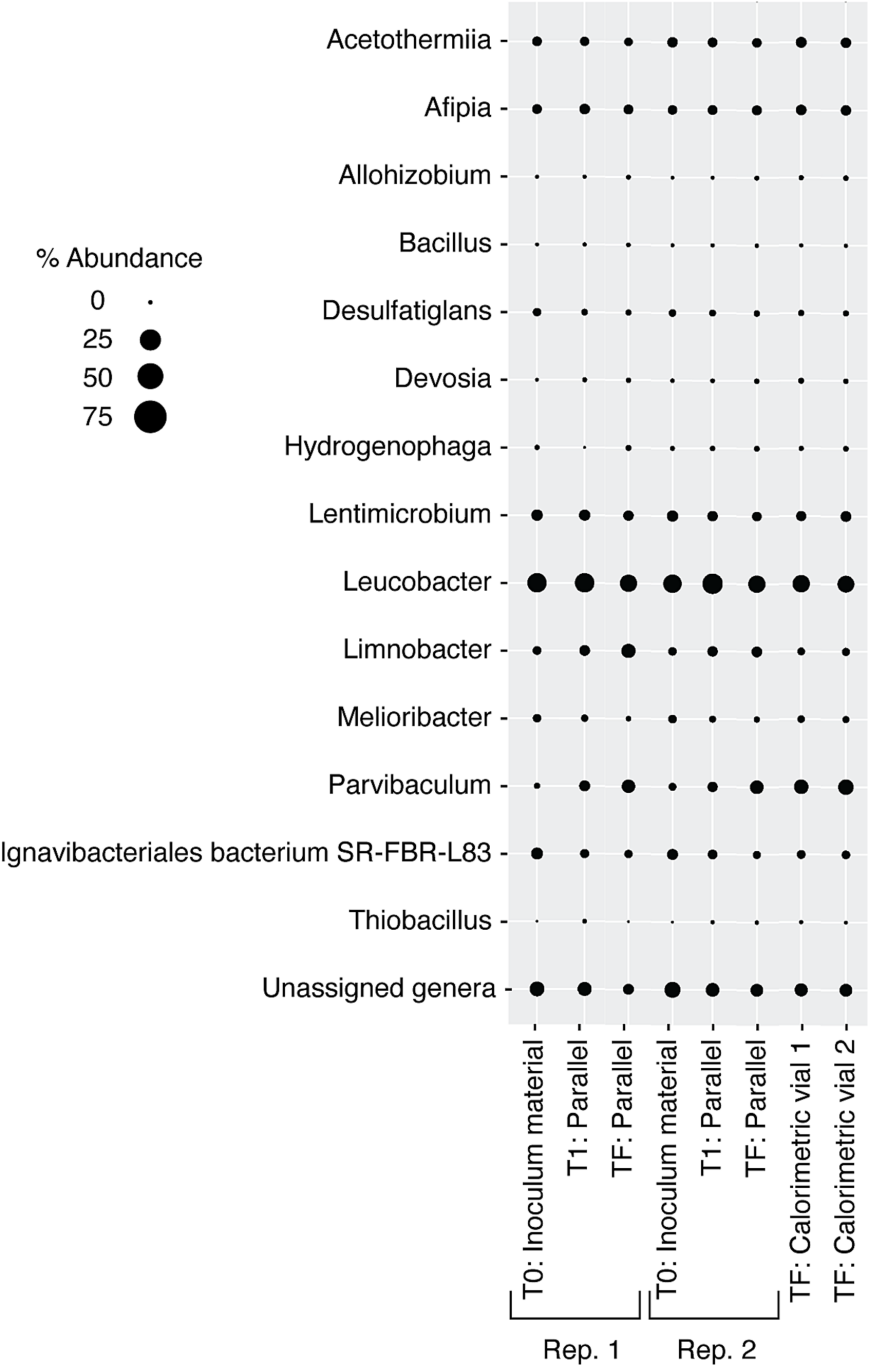
Relative abundance of microbial genera in DeMMO 6 fluids before (*t_0_*) and after (*t_F_*) and 46 h into (*t_1_*) incubation with formate. “Parallel” refers to duplicate incubation experiments run outside the calorimeter, and these parallel experiments were replicated (Rep. 1 and Rep. 2). Replicate experiments run inside the calorimeter are noted as “Calorimetric vial 1” and “Calorimetric vial 2”. “Unassigned genera” indicates taxa for which taxonomy could not be assigned at the genus level.

The microbial community in the formate addition cultures remained markedly consistent, with almost no variation in structure between any replicates or time points (Figure 8). In both *t_0_* replicates, the dominant community member was *Leucobacter* (~25% relative abundance), with all other community members present in less than 10% abundance. The community structure after 46 h, *t_1_*, looks very similar to the community at *t_0_*, with a slight increase in the abundance of *Parvibaculum* and a slight decrease in the abundance of *Ignavibacteriales* bacterium SR-FBR-L83. These trends stayed consistent after 336 h, *t_F_*, across all replicates within and outside of the calorimeter. *Leucobacter* continued to dominate the community at ~25% abundance, but a larger percentage of the community was composed of *Parvibaculum* (~10%).

While formate additions resulted in an increase in cell density and a change in heat flow, acetate had a negligible effect on the community, even when combined with nitrate. Simultaneous addition of acetate and nitrate to borehole waters resulted in ~175 nW stronger heat signal than unamended groundwater within 100 h (Figure 3). Over the course of the experiment, nitrate and acetate concentrations remained unchanged at ~10 mM each, while cell densities increased variably from 4.74 × 10^4^ cells/mL to a maximal density of 1.20 × 10^6^ cells/ml at 216 h (Supplementary Figure 4; Figure 3). In all inorganic EA and organic ED amendment experiments, heat signals were < 500 nW, with the majority closer to ~100 nW at 100 h.

#### 3.2.2 Amino acids

All amino acid-amended borehole fluids produced a substantial and highly replicable heat flow signal whose maximal magnitude correlated with amino acid concentration. Amendments with 0.3710 mM casamino acids resulted in a maximal heat flow of ~2.25 μW at ~32.5 h (Figure 4C). Heat flow for the next highest concentration of amino acids, 0.0834 mM (red curve), peaked at ~1.1 μW, approximately 5 h after the 0.3710 mM amendment. The heat flow signal for 0.0675 mM (purple curve) reaches the same peak as that for 0.0834 mM amino acids, but tapers for twice as long. The maximal heat signal for the 0.0237 mM amendment (blue curve) is less than those for 0.0675 mM and 0.0834 mM, but the tapering ends at a similar time as it does for the 0.0675 mM experiment. Amendment with 0.0093 mM amino acids results in a much lower heat flow than those experiments with higher amino concentrations, maximally at ~0.2 μW heat flow.

In many of the amino-amended heat flow signals, a small peak occurs around 10 h, followed by a much larger peak around 30–40 h (Figure 4A). The sharpness of the large peak was directly correlated to amino acid concentration, with higher concentrations resulting in steeper peaks. From the maximal heat flow at the peak, all signals dropped off between ~35 and ~ 55 h, except in the case of 0.0093 mM amino acid amended fluids.

The total heat produced during an amino acid addition experiment was influenced by the length of time that the heat flow signal tapered after the steep drop-off following the peak (Figure 4B). For instance, the largest total heat signal, 0.07 J, was present for 0.0675 mM (Figure 4D) which had the same peak width as the 0.0237 mM experiment (Figure 4A). However, the sample with 0.0675 mM casamino acids had a larger maximal heat prior to the tapering period (~1.1 vs. ~0.75 μW). Little heat production occurred after 45 h in most amended samples.

**Figure 9:**
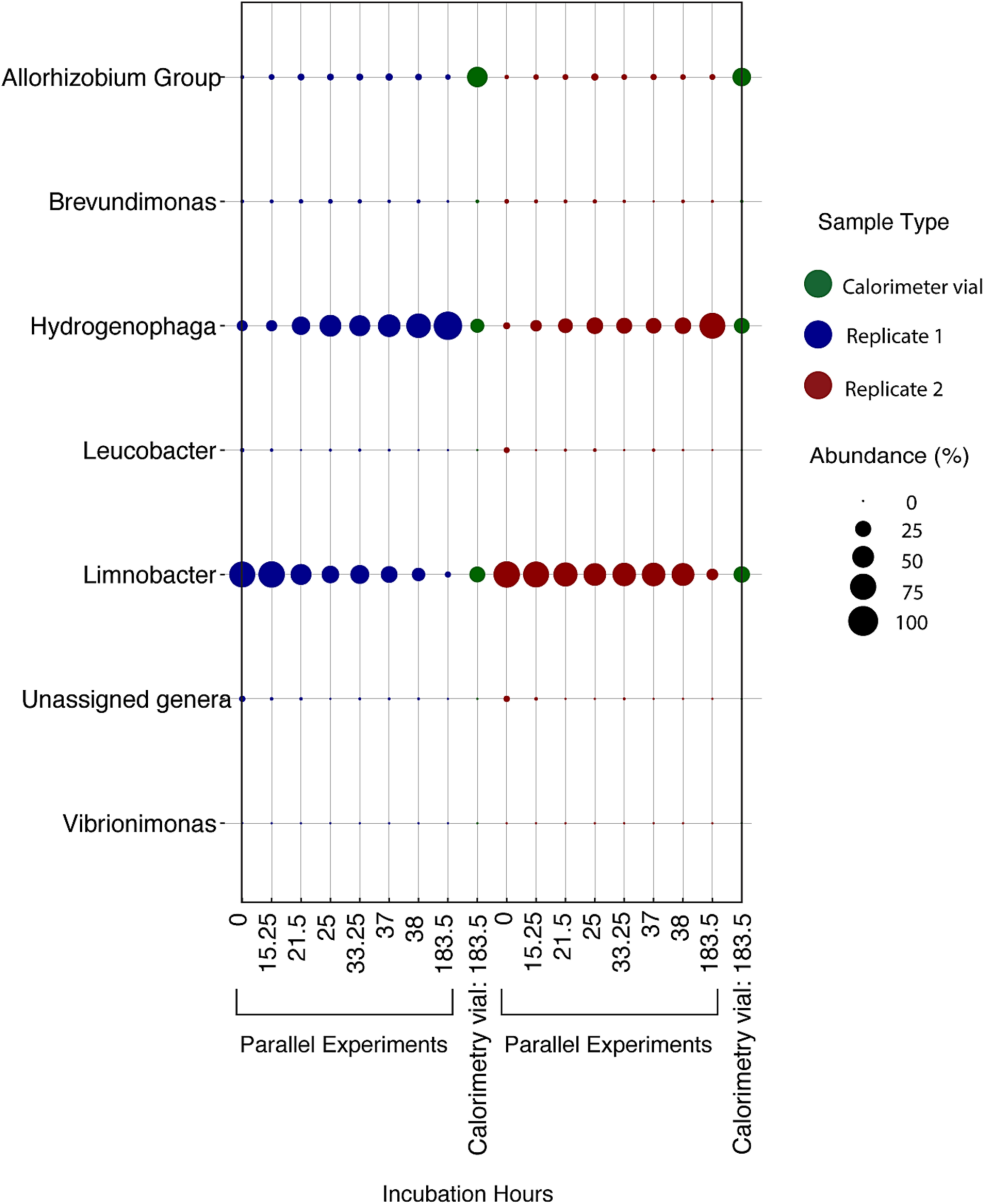
Relative abundance of microbial genera in DeMMO 6 fluids amended with 0.371 mM amino acids over various times throughout 183.5 h in two experiments incubated outside of the calorimeter (“parallel experiments”) and two replicates incubated inside of the calorimeter (“calorimetry vial”). Calorimetry vial samples were collected at 183.5 h. “Unassigned genera” indicates taxa for which taxonomy could not be assigned at the genus level.

Over the duration of the amino acid amended experiments, the community was dominated by two major taxa that are endemic to the SURF community: *Hydrogenophaga* and *Limnobacter* (Figure 9). In addition to these two taxa, *Allorhizobium* became a major part of the community in the experiments taking place inside the calorimeter. The addition of amino acids slowly changed the community over time, with *Limnobacter* dropping off in abundance from as much as ~75% to as little as ~5%. *Hydrogenophaga* was present at some abundance in all the collected DeMMO 6 groundwater, although it particularly thrived when exposed to more complex organic carbon sources, becoming as much as 75% of the community during amino acid addition. Figure 5 depicts the growth curves corresponding to these data, indicating a cell density increase in the community from 1.16 × 10^5^ cells/mL to 1.41 × 10^8^ cells/mL within 70 h. During this time, sulfate concentrations did not change within the margin of error, while nitrate and oxygen concentrations remained below the detection limit (Supplementary Figure 5). On average, carbon dioxide rose from 1.40 × 10^−8^ mol/mL to 2.66 × 10^−7^ mol/mL while hydrogen dropped from 1.03 × 10^−6^ mol/mL to 2.58 × 10^−7^ mol/mL.

To test whether the amino acid addition signal results from microbes splitting the ammonia group off the amino acids, amino acids were amended into DeMMO 6 fluids at 0.371 μM and 9.3 μM concentrations. Additionally, 1 M ammonium was added to samples at each of these amino acid concentrations. Only minimal differences were noted between samples amended with amino acids vs. samples amended with amino acids and ammonium (Supplementary Figure 6).

## 4 Discussion

### 4.1 *In-situ* activities are low throughout SURF

The investigation into microbial metabolic activity within the SURF borehole fluids from DeMMO 6 reveals a distinct response to nutrient amendments, as evidenced by measured variations in heat flow. Specifically, introducing small concentrations of amino acids (0.3710 mM) stimulated a metabolic reaction that led to the generation of approximately 2.2 μW within a span of 25 h. This indicates a pronounced microbial response to the added substrates, translating into an observable thermodynamic effect. Similarly, the addition of formate and a nitrate/acetate mixture induced significant variations in heat flow, underscoring a substantial metabolic shift compared to the baseline established by unamended borehole fluids. Contrarily, the addition of other substrates such as citrate, lactate, pyruvate, and acetate (in the absence of nitrate) did not manifest in a marked increase in heat flow, suggesting a selective metabolic responsiveness to nutrient types. Despite this apparent lack of response, the results pertaining to these substrates are consistent and exceed the calorimeter’s threshold of detection, validating their reliability. Additionally of interest, aging of samples in the laboratory did not result in differences in the maximum heat flow a sample could obtain, but did change the amount of time required for a sample to obtain this heat flow signal. This is evident when comparing the nitrate and acetate co-amendment data in Figures 1, 3, which represent data collected, respectively, within weeks of the sample being collected, and after 3 months. In contrast, unamended fluids from the remaining five DeMMO boreholes failed to yield replicable outcomes, likely attributable to diminished cell densities and inherently low microbial activity within those environments. DeMMO 6 may have increased cell densities and activities relative to the other 5 boreholes resulting from being capped with a steel manifold which inhibits flow. This differential response underscores the complexity of microbial communities’ metabolic dynamics and highlights the potential of specific substrates to activate or enhance microbial processes within subsurface ecosystems.

The SURF VPR is another indicator of low microbial activity at this site. Active microbial communities tend to be characterized by a standard ratio of viruses to prokaryotes of about 10:1 (Thingstad, 2000). This ratio is based on the notion that viruses cannot replicate unless their hosts are sufficiently active to utilize their replication machinery. Based on previous studies examining VPR across a variety of environments, the average VPR from SURF, 2.03, is very low. For instance, the average VPR in deep sea environments is 28.45 and for estuaries, it is 11.35 (Parikka et al., 2017). The lowest reported average values across an environment are from waterflow or rivers, at 9.38, although the lowest VPR yet reported (0.0001) was found in deep marine sediments (Yanagawa et al., 2014). These low VPR values may indicate a cost to phage activity, wherein low metabolic rates become necessary for survival *in-situ* (Thingstad et al., 2014). Regardless of the cause of low metabolic rates at SURF, the VPR present across the SURF boreholes is indicative of a dormant or near-dormant metabolic state in the site fluids. It is likely that cell densities and activities are higher in fissures in the source rock, where mineral-associated biofilms provide more hospitable conditions for microbial life (Casar et al., 2021a). However, in the fluids, the case for an inactive microbial community is also supported by the lack of extractable mRNA across all DeMMO boreholes. This trend is of particular interest given that most microbial modeling assumes that microbes are either dead or alive and active, when in fact a state of maintenance may be more applicable in energy-limited systems (Bradley et al., 2018). This study supports the notion of a population that is engaged in very low-energy maintenance activities and provides a variety of potential methods for assessing metabolic rate during dormancy.

The low heat flow rates noted in DeMMO borehole fluids are conspicuously less than those reported for bacteria grown in media but are consistent with environmental values estimated through modeling. Using a reaction transport model and Gibbs energy calculations, it can be determined that, extremely energy-limited cells can exist on 1.5×10^−8^ pW (Bradley et al., 2020). While the enthalpy-based measurements of power employed in this study do not indicate such low activity, heat flow also does not reach the levels described in more active communities. For context, a pure culture of *Shewanella oneidensis MR-1* grown under oxygen limitation maximally produces a heat flow of ~24 μW (Robador et al., 2018), a value that is 48 times greater than the maximal heat flow resulting from the SURF borehole water even after amendment with high concentrations of organic acids and EAs. For DeMMO 6, unamended samples produced 100 nW for 5.2 × 10^5^ total cells, indicating that a single cell under those conditions uses around 0.19 pW. This value is in a similar range to that for a single cell in soil (1 pW to 10^−5^ pW), and in the basaltic crust (5.68 pW), and is well above the estimated range for a single cell in marine sediments (10^−4^ pW to 10^−8^ pW; Robador et al., 2016; Bay et al., 2021; Bradley et al., 2022; Zhao et al., 2021), which are based on Gibbs energies, not enthalpies, of reaction. The most straightforward explanation for why consistent heat signals could not be obtained for the other boreholes is that the cell density at those boreholes was too low to generate a replicable signal. The DeMMO 1–5 borehole fluids contain about to a 10^3^ cells/mL. Assuming the 0.192 pW of power from DeMMO 6 holds true for the other boreholes, the heat flow for DeMMO 1, at 1.607 × 10^3^ cells/mL would be 0.31 nW. This value is below the detection limit of the calorimeter.

### 4.2 Energetically favorable or genomically encoded does not mean active

This study tested some of the predictions made by previous papers predicting potential metabolisms at this site (Osburn et al., 2014; Momper et al., 2017; Momper et al., 2023). Nitrate reduction pathways are present at DeMMO 6 and the use of this EA is energetically favorable given the conditions present at this borehole. Although the addition of nitrate to DeMMO 6 borehole fluids did result in a larger heat flow signal than was present in unamended fluids and promoted cell replication, it failed to produce the large microbially-produced heat signal seen with amino acid addition. Sulfate reduction pathways are also both energetically favorable and commonly encoded at DeMMO 6, but sulfate is abundant at high concentrations (~45 mM) on site, and the supplementation of an additional 10 mM sulfate in calorimetric experiments did not impact heat flow in this study (Supplementary Figure 7).

It has been proposed that organic acids are being produced in the deep subsurface biosphere by fermentative bacteria, and that these organic acids are in turn utilized by organotrophs (Teske, 2005). Acetate has also been shown to stimulate growth in deep subsurface fluid communities from Finland (Miettinen et al., 2018), and genomic studies of other deep subsurface sites have reported abundant genes for heterotrophic processes (Nyyssönen et al., 2014; Nuppunen-Puputti et al., 2022). In the SURF system, acetate, as a proxy for organic matter, was demonstrated to be the most exergonic ED (Osburn et al., 2019). Additionally, genes for acetate and formate oxidation are abundant across the six DeMMO boreholes (Momper et al., 2023). In this study, acetate amendment had a greater impact when co-amended with nitrate, potentially indicating microbial denitrification on site is controlled by the availability of organic acids like acetate. Formate amendment resulted in greater overall heat flows than acetate amendment, although uniquely, this organic acid resulted in an oscillating heat flow indicating potential metabolic shifts. The increase in cell density but lack of concurrent change in community structure noted during this amendment could indicate that all community members are equally sensitive to formate, but another possibility is that the microbes in this community are not doubling so much as becoming more metabolically active and therefore easier to detect. A limit to this study is that all conditions were uniformly tested at a 10 mM concentration, which was selected after lower concentrations of acetate (~1 mM) failed to produce a measurable signal (data not shown). However, 10 mM concentrations of may have introduced inhibitory effects or altered microbial activity patterns. From the previous studies of the genomic potential at SURF, it is difficult to determine which of the many genes for the use of small organic molecules present at DeMMO 6 are actively being transcribed given the environmental conditions the community is experiencing (Momper et al., 2023). The data in this study indicate that organic acids appear to have a lesser impact on catabolic processes than amino acids.

### 4.3 Deep continental subsurface life is uniquely poised for amino acid metabolism

The amendment of even very low concentrations of casamino acids (0.0093 mM) resulted in an increase in heat flow, total heat, and cell density above that in unamended fluids. A higher amino acid concentration did not necessarily equate to greater total heat. Rather, samples with less amino acids produced a lower but more prolonged peak that produced more total heat. Among the microbial taxa changing most strongly in response to the amendment of amino acids were *Hydrogenophaga* and *Limnobacter*, which were present *in-situ* at the time of sampling and therefore unlikely to be contaminants. In fact, *Hydrogenophaga* was the dominant microbe in the original rock core removed to prepare the DeMMO 6 borehole (Momper et al., 2017), is commonly observed throughout gold mine and continental subsurface environments (Santini et al., 2002; Baker et al., 2003; Nuppunen-Puputti et al., 2022) and has been grown under anaerobic conditions (Duffner et al., 2022). Further, although the marker-gene based community composition of the starting inoculum materials differ slightly between experiments, this is most likely due to the inherent stochasticity of energy-limited deep subsurface environments and is likely to represent the variability that is naturally present in these environments. The preferential growth of *Hydrogenophaga* and other known deep subsurface organisms following nutrient amendment indicate that their responses are relevant to the *in-situ* environment. Although members of the Candidate Phyla Radiation (CPR) group were not reported in this study, it is possible that they are also present in the samples due to the difficulty with which they can be resolved via 16S rRNA analysis.

Growth of the DeMMO 6 communities resulting from amino acid addition could be due to nitrogen limitation, which this study tested with 1 M additional NH_4_ supplementation. Although these conditions are not intended to represent a natural environment, they did indicate that additional ammonium failed to produce additional heat signal. Future studies would benefit from supplementation of ammonium at more environmentally relevant concentrations, including during the addition of other substrates. Ammonium has been indicated to be the major source of nitrogen in deep subsurface communities and is generally assimilated via the amino acids glutamine and glutamate (Nyyssönen et al., 2014). At DeMMO 6, the genes for ammonium oxidation are a minor component of the site genomic potential, with oxidative nitrogen metabolisms being rarely encoded across all DeMMO boreholes (Momper et al., 2023). The results of this study suggest that in the conditions tested, microbial activation and the subsequent metabolic processes, as indicated by heat production, are not directly facilitated by or dependent on ammonium as a metabolic substrate.

The Stickland reaction, or the fermentation of amino acids, is a potential catabolic process that could explain the formation of the observed heat flow. However, during the production of 0.0433 J of heat, this reaction produces 3.53 × 10^−7^ moles of CO_2_, an order of magnitude higher than the quantity of CO_2_ measured throughout these experiments. It is therefore likely that if the Stickland reaction is being employed by the SURF community, either a portion of the CO_2_ is being utilized by the community upon formation (e.g., for biomass production), is being absorbed by the environment (e.g., via carbonate formation), or a secondary reaction is resulting in a proportion of the heat production. These potential secondary reactions include the oxidation of amino acids with NO_3_^−^, a process that would produce more CO_2_ than was measured following these incubations. Moreover, NO_3_^−^ in any concentration was not detected throughout the amino acid addition experiments. The reduction of O_2_ with amino acids has the potential to explain the measured heat flow signal, producing approximately the observed amount of CO_2_. Throughout these experiments, however, O_2_ remained below the detection limit, although only a very low quantity of O_2_ (lower than the detection limit of our gas chromatograph) would be required to produce this heat flow signal. The sulfide present in DeMMO 6 fluids is likely to reduce any O_2_ originally present in the sample, although sulfide was lower than usual in these samples at the time of collection (Table 1; Momper et al., 2023). Potential evidence in support of the use of O_2_ comes from the taxa present in the amino acid amended fluids, which hosts genera containing predominantly aerobic or microaerobic species. It is possible that the *Hydrogenophaga* in the fluids preferentially reduces O_2_ with *in-situ* H_2_ or organic matter, and during times of O_2_ and organic matter starvation, goes dormant. Additional community members could be engaged in sulfate reduction with H_2_. The latter metabolism would consume 7.91 × 10^−7^ moles of H_2_, which is similar to the 6.11 × 10^−7^ moles of H_2_ consumed throughout these experiments, while sulfate reduction with amino acids is unlikely, as this produces 7.93 × 10^−6^ moles of CO_2_, substantially more than what was produced during sulfate addition test work performed during this experiment (Supplementary Figure 7).

Due to the volumes of H_2_ consumed and CO_2_ produced in SURF fluids following amino acid addition, it seems likely any trace amounts of oxygen were initially consumed by the SURF community alongside amino acids, and perhaps heavily contributing to the measured heat flux. It is worth noting that the trend wherein larger amounts of amino acids produced a sharper heat flow spike, but less total heat production, supports the concept of a community wherein a small quantity of oxygen plus an abundance of amino acids allowed for a spike in activity. When less amino acids were present, the heat signal reached a lower but more sustained peak over time, indicating that a limiting reactant to growth – or potentially simply other metabolic activities – was not so readily exhausted. Perhaps in these experiments and following oxygen exhaustion in the higher concentration amino acid addition experiments, the community shifted toward sulfate reduction with hydrogen, thus explaining the quantity of hydrogen that was consumed over time. The genomic potential for oxygen, hydrogen, and sulfate utilization have all been reported for the DeMMO 6 borehole (Momper et al., 2023). Sulfate reduction is a dominant metabolism in the deep subsurface (Amend and Teske, 2005), and hydrogen is considered a major driver of many subsurface communities (Nealson et al., 2005). At DeMMO 6, the high concentration of sulfate complicated precise measurements of microbially-mediated changes in sulfate (Supplementary Figure 7), and it is possible that taxa with known sulfate reduction metabolisms did not dominate the community 16S rRNA data due to the genes for sulfate reduction being sequestered in CPR taxa, which are not well-resolved via 16S. While no significant consumption of sulfate was noted, the inclusion of amino acids was nonetheless necessary for the large concentration of *in-situ* sulfate to be used.

Future calorimetric work could test the combinative impact of amino acid addition with other substrates, such as organic acids or additional sulfate. Similar studies would also benefit from considering other compounds that are present in deep subsurface environments. Microbial utilization of the sulfur compounds thiosulfate and tetrathionate was investigated via calorimetry in Wentzien et al., 1994, and study of these compounds, in addition to elemental sulfur, helps solidify the story of sulfur cycling at deep subsurface sites. Additionally, it should be noted that calorimetry is occasionally used to assess the degradability of various ores via bioleaching pathways (Schroter and Sand, 1993; Schippers et al., 1995), and as such, future studies would benefit from conducting experiments using solid minerals, such as pyrite. Calorimetric studies investigating the microbial oxidation of pyrite using canonical bioleaching taxa have produced strong signals (Rohwerder et al., 1998), and previous studies from SURF have indicated that pyrite bioleaching may play a significant role in influencing the fluid chemistry onsite (Casar et al., 2021a). Lastly, methane or hydrogen addition would be a particularly attractive target for future work, but precise gas addition was not possible in this experiment without custom modifications to prevent leakage. Based on this study, amino acid addition, whether as a specific organic carbon source or as a building block for proteins, is recommended as a trigger for activity in this community regardless of other nutrient additions.

## 5 Conclusion

Like many deep continental subsurface environments, SURF appear to exhibit exceptionally low activity. This conclusion is drawn from several observations: only minimal amounts of RNA are transcribed, Virus-to-Prokaryote values suggest cells are in a non-growth state, and ultra-sensitive calorimetry fails to detect any heat signals from catabolic reactions, even after the addition of substrates capable of yielding redox energy. Altogether, these findings indicate a microbial community in a state of dormancy. Despite the low basal activity levels, microorganisms in SURF fluids demonstrate viability, as evidenced by their pronounced metabolic response to casamino acids and more tempered reactions to formate, acetate, and nitrate. This indicates not only the presence of life but also a selective responsiveness to different chemical amendments, highlighting the potential for metabolic activation under specific conditions.

The ability to maintain a low level of activity in a state akin to dormancy follows studies suggesting that in nutrient-limited environments, the fittest organisms in a community are those that can survive times of nutrient limitation, not those which can grow well (Bradley et al., 2019). By characterizing quantitatively and qualitatively the total heat evolved throughout calorimetric experiments, we have shown that the addition of amino acids to the SURF microbial community stimulates microbial growth and metabolic activity. Although samples amended with the highest concentration of amino acids exhibit the highest peak heat flow, it is the samples with a lower amino acid concentration that generate the greatest total amount of heat. Given this trend, measurements of reactants and products, and our calculations of the enthalpy of potential reactions, it is likely that any oxygen is rapidly used in this environment when amino acids are also present. Amino acid addition also appears to allow for the engagement of other catabolic processes, perhaps sulfate reduction with hydrogen or the Stickland reaction. The triggering of metabolic processes following amino acid addition in conjunction with the low heat flow data prior to nutrient amendment indicates that the SURF community is in a form of maintenance until the necessary conditions become available.

Amino acid limitation is commonly due to a lack of microbial amino acid biosynthesis during times of low energy flux (Payne and Wiebe, 1978), a state that certainly describes SURF fluids. Amino acid limitation has been noted in other oligotrophic environments, e.g., in coastal lagoons (Cotner et al., 2000), the subarctic Pacific (Kirchman, 1990), and in the Southern Ocean (Church et al., 2000). Yet, the role of amino acid limitation as a regulatory factor for life in the continental subsurface has not been previously identified.

## Data Availability

The original contributions presented in the study are publicly available in the NCBI repository. This data can be found here: https://www.ncbi.nlm.nih.gov/bioproject, accession number PRJNA1196095.
