# Supplementary material for: Calorimetric measurement of energy and nutrient stimulation of microorganisms from the continental deep subsurface
Source: Front Microbiol. 2024 Dec 23;15:1455594. doi: 10.3389/fmicb.2024.1455594 (PMC11701026; doi:10.3389/fmicb.2024.1455594)
Supplement: Supplementary file 1 [file Supplementary_file_1.docx]

The following figures and tables accompany the text “Calorimetric measurement of energy and nutrient stimulation of microorganisms from the continental deep subsurface” and describe information that is supplemental to the main manuscript.

**Supplemental Figure 1.** Heat fluxes (Power) as a function of time for the experiments in which amino acids of differing concentrations were added to borehole fluids from DeMMO 6. Pink lines represent raw data recorded by the calorimeter and black dashed lines correspond to the heat flux associated with inserting the samples vials into it, as determined by a power law fit, more details of which are available in the “Methods” section. Numerical values of heat and power reported throughout this manuscript were determined by subtracting the signals associated with the black lines from the pink lines.

**

**Supplemental Figure 2.** Cell densities as a function of time in DeMMO 6 borehole fluids amended with 10 mM each of citrate, lactate, and pyruvate.


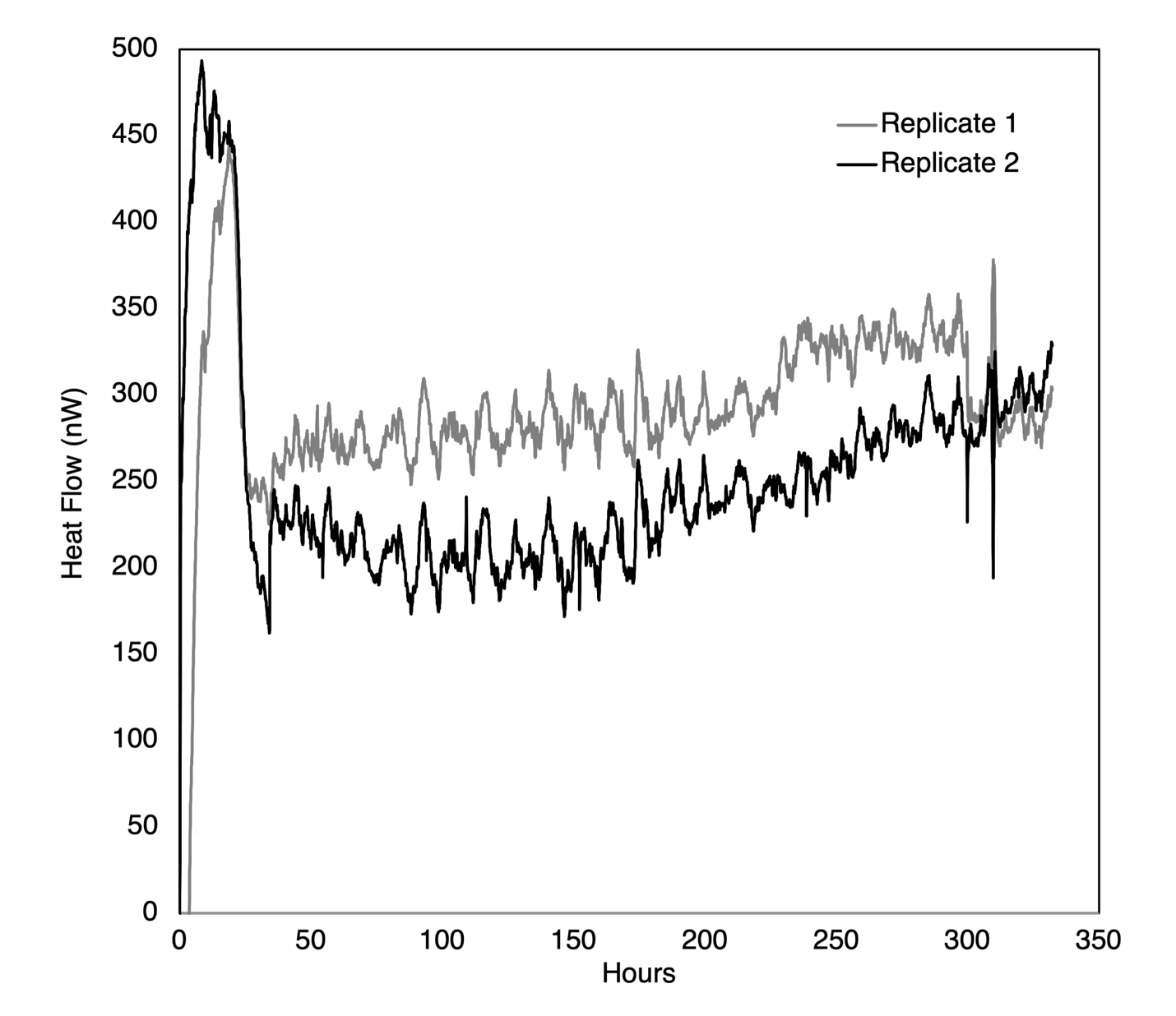


**Supplemental Figure 3.** Heat flow (nW) as a function of time for two replicate amendment experiments in which formate (10mM) was added to borehole fluids from DeMMO 6. The baseline heat flow signal has been corrected using the synchronization method.

**

**Supplemental Figure 4.** Acetate concentrations over time in DeMMO 6 borehole fluids amended with 10 mM each of acetate and nitrate.

*
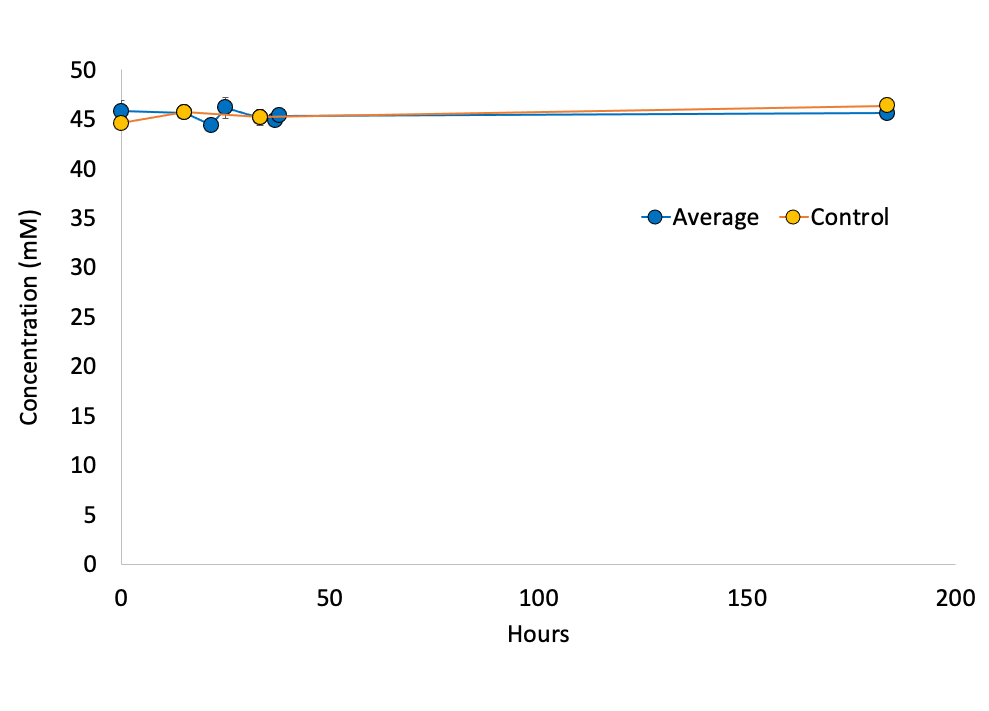
*

**Supplemental Figure 5.** Average sulfate concentration over time in DeMMO 6 borehole waters amended with 0.371 mM amino acids, relative to a killed control.

Supplemental Figure 6. Heat flux (Power) over time in DeMMO 6 fluids amended with 1 M NH_4_ and 9.3 μM amino acids (A) and 1 M NH_4_ and 0.371 mM amino acids (B). In both plots, the experiment is replicated at the same amino acid concentration, but without 1 M NH_4_.

*
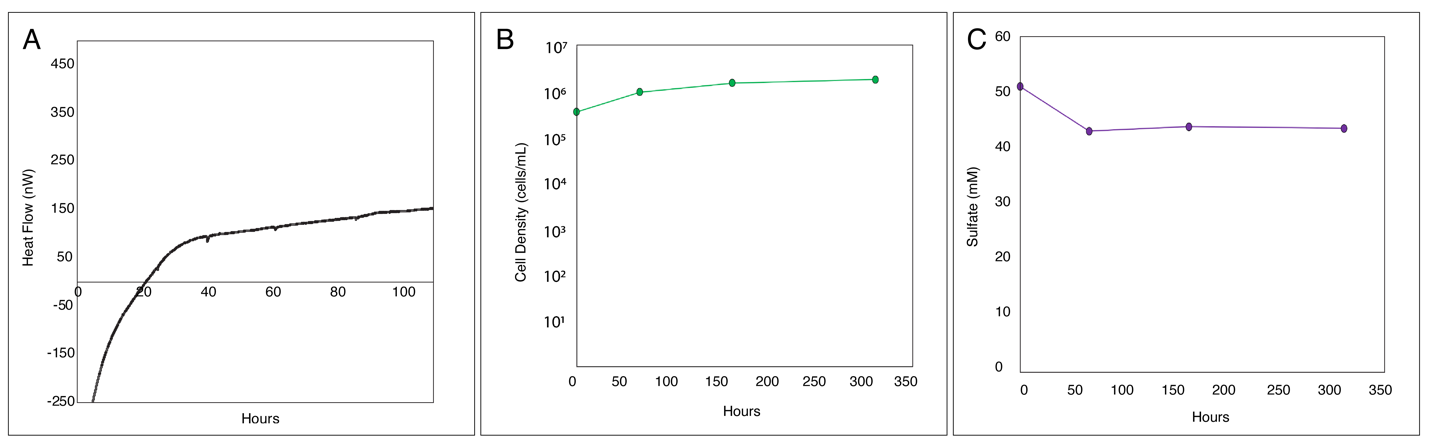
*

**Supplemental Figure 7.** (A) Heat flow arising from DeMMO6 fluids amended with 10 mM sulfate. (B) Cell density as a function of time DeMMO6 borehole fluids amended with sulfate (10 mM). (C) Sulfate concentration as a function of time in DeMMO 6 borehole fluids supplemented with an additional 10 mM of sulfate.
